# Comparison of phenology and pathogen prevalence, including infection with the *Ehrlichiamuris*-like (EML) agent, of *Ixodes scapularis* removed from soldiers in the midwestern and the northeastern United States over a 15 year period (1997-2012)

**DOI:** 10.1186/s13071-014-0553-z

**Published:** 2014-12-04

**Authors:** Ellen Stromdahl, Sarah Hamer, Sarah Jenkins, Lynne Sloan, Phillip Williamson, Erik Foster, Robyn Nadolny, Chad Elkins, Mary Vince, Bobbi Pritt

**Affiliations:** U.S. Army Public Health Command, Aberdeen Proving Ground, MD USA; Texas A&M University, College Station, TX USA; Mayo Clinic, Rochester, MN USA; University of North Texas Health Science Center, Ft. Worth, TX USA; Creative Testing Solutions, Tempe, AZ USA; Michigan Department of Community Health, Lansing, MI USA; Department of Biological Sciences, Old Dominion University, Norfolk, VA USA

**Keywords:** Climate change, Co-infection, *Ehrlichia muris*-like agent, Engorgement, *Ixodes scapularis*, Pathogen prevalence, Phenology, Surveillance, Tick population establishment

## Abstract

**Background:**

Since 1997, human-biting ticks submitted to the Department of Defense Human Tick Test Kit Program (HTTKP) of the US Army Public Health Command have been tested for pathogens by PCR. We noted differences in the phenology and infection prevalence among *Ixodes scapularis* ticks submitted from military installations in different geographic regions. The aim of this study was to characterize these observed differences, comparing the phenology and pathogen infection rates of *I. scapularis* submitted from soldiers at two sites in the upper Midwest (Camp Ripley, MN, and Ft. McCoy, WI) and one site in the northeastern US (Ft. Indiantown Gap, PA).

**Methods:**

From 1997 through 2012, the HTTKP received 1,981 *I. scapularis* from the three installations and tested them for *Anaplasma phagocytophilum*, *Babesia microti*, *Borrelia burgdorferi* and the *Ehrlichiamuris*-like (EML) agent using PCR; pathogen presence was confirmed via sequencing or amplification of a second gene target. Pathogen and co-infection prevalence, tick engorgement status, and phenology were compared among installations.

**Results:**

Greater rates of *A. phagocytophilum* and *Ba. microti* infections were detected in ticks submitted from installations in Minnesota than in Wisconsin or Pennsylvania, and the EML agent was only detected in ticks from Minnesota and Wisconsin. Midwestern ticks were also more likely to be co-infected than those from Pennsylvania. Both adult and nymphal ticks showed evidence of feeding on people, although nymphs were more often submitted engorged. Adult *I. scapularis* were received more frequently in June from Minnesota than from either of the other sites. Minnesota adult and nymphal peaks overlapped in June, and submissions of adults exceeded nymphs in that month.

**Conclusions:**

There were clear differences in *I. scapularis* phenology, pathogen prevalence and rates of co-infection among the three military installations. Seasonal and temperature differences between the three sites and length of time a population had been established in each region may contribute to the observed differences. The synchrony of adults and nymphs observed in the upper Midwest has implications for pathogen infection prevalence. The EML agent was only detected in Minnesota and Wisconsin, supporting the previous assertion that this pathogen is currently limited to the upper Midwest.

## Background

*Ixodes scapularis* is an important vector for several human pathogens, including *Borrelia burgdorferi*, *Anaplasma phagocytophilum*, *Babesia microti*, and the newly-described *Ehrlichia muris*-like (EML) agent [1], agents of Lyme disease, anaplasmosis, babesiosis, and ehrlichiosis, respectively. In order to facilitate the care of military personnel, dependents and Department of Defense (DOD) civilian employees that have been exposed to *I. scapularis* and other ticks, the US Army Public Health Command (USAPHC) developed the DOD Human Tick Test Kit Program (HTTKP) in 1989 which identifies ticks removed from these individuals and tests them for associated pathogens [2]. Results of tick species identification, tick engorgement status and pathogen analysis are reported to the health care provider for the individual reporting the tick bite in order to aid in the assessment of tick-borne disease risk. The results of tick identification and pathogen analyses are also used in combination with US Army entomological field studies (field-collected tick and mammal host infection frequencies) to assess the threat of tick-borne diseases on specific military installations and guide decisions for tick-borne disease management based on national guidelines and published studies [3,4]. Starting in 1997, the HTTKP used polymerase chain reaction (PCR) to test ticks for human pathogens. From 1997-2012, 40,917 ticks (26,613 *Amblyomma americanum*, 7,193 *Dermacentor variabilis*, 6,860 *I. scapularis* and 251 other spp.) were received and tested from approximately 100 military installations primarily in the eastern half and upper midwestern section of the US. During this time, several differences in the phenology and pathogen prevalence among *I. scapularis* from different geographic regions were noted based upon tick submissions. Specifically, the adult life stage of *I. scapularis* was received more frequently in summer months (June and July) from military medical treatment facilities in the upper midwestern US than from military installations in other regions that routinely submit ticks, and the EML agent was only detected in *I. scapularis* removed from soldiers at Camp Ripley, MN and Ft. McCoy, WI and not in *I. scapularis* from any other military installations within the geographic range of the tick, despite the testing of >2,000 *I. scapularis* ticks from all areas (E.S., unpublished data).

The aim of this study was to further characterize these observed differences, comparing the phenology and prevalence of *A. phagocytophilum*, *Ba. microti*, *B. burgdorferi* and the EML agent in *I. scapularis* submitted from soldiers at two sites in the Upper Midwest, Camp Ripley, MN, (46°10’32”N, 94°22’23”W), and Ft. McCoy, WI, (44°0’36”N, 90°40’60”W), and one site in the northeastern US, Ft. Indiantown Gap, PA (40°26’13”N, 76°34’34”W), from 1997 through 2012. These three sites were chosen because they share several important environmental features: all comprise large acreages of brush and woods that provide excellent habitat for ticks and their vertebrate hosts, notably *Peromyscus leucopus*, the white-footed mouse, an important reservoir of several human pathogens including *A. phagocytophilum*, *Ba. microti*, *B. burgdorferi* and the EML agent. The distribution of tick species there are very similar. The dominant anthropophilic tick species are *I. scapularis* and *Dermacentor variabilis*, and they are very abundant at the three installations. Interestingly, sympatric *A. americanum* populations have not been reported from these installations [5,6], and military personnel from these locations have not submitted this species to the HTTKP (E.S., unpublished data). At each site, the predominant tick-borne diseases are Lyme and anaplasmosis. In addition, soldier populations at the three installations are standardized in several ways that facilitate comparisons. All three installations are field training facilities used primarily by Reserve and National Guard units that deployed there for 2-wk sessions. During deployment periods, soldiers left the installations infrequently and dressed in identical uniforms while training. The ticks submitted to the HTTKP were from these trainees and rarely from dependents or DOD civilians. Only ticks that were attached to humans were submitted. All soldiers were instructed to use insect repellents and received similar training for personal tick surveillance. The HTTKP is a long-standing and well-utilized program at all three installations, and the regulations controlling and standardizing soldier activities at these field-training installations enabled us to have a more precisely regimented human population, which is unique and valuable for passive tick surveillance studies.

## Methods

### Morphologic assessment of ticks

*I. scapularis* removed from military personnel and submitted to the HTTKP from Camp Ripley, Ft. McCoy and Ft. Indiantown Gap from 1997 through 2012 were identified using standard taxonomic keys. Only ticks that had actually bitten humans were accepted by the HTTKP. Only female adult and nymphal *I. scapularis* totals were included in this study since they comprise the vast majority (>98%) of *I. scapularis* submitted to the HTTKP. The engorgement status of each female and nymphal tick was estimated as unengorged, partly engorged, or fully engorged at the time of examination. The date of tick removal reported by the patient and health care provider on the submission form was used as a surrogate marker for when the tick was active. Ticks were only included in the phenology comparison if the date of tick removal was available.

### DNA isolation

From 1997 through 2004, DNA isolation was performed using the IsoQuick nucleic acid extraction kit (ORCA Research, Bothell, WA) as described previously [2]. Beginning in 2005, the Zymo Genomic DNA II Kit (Orange, CA) was adopted to improve workflow while maintaining DNA quality and quantity [7].

### PCR

Individual *I. scapularis* removed from soldiers were tested for *A. phagocytophilum, Ba. microti, B. burgdorferi* and EML using PCRs as described below. All initial positive results were confirmed by testing the DNA extract with the same PCR or second PCR for a different genetic region (Table 1), or DNA sequencing for selected pathogens. Positive specimens were defined as samples that produced at least two separate PCR positive results.

**Table 1:** **PCR primers for human pathogens associated w.** ***Ixodes scapularis*** **ticks, DOD Human Tick Test Kit Program**

**Pathogen**	**Year**	**Initial screen**	**Reference**	**Reconfirmation** ^**a**^	**Reference**
***Anaplasma phagocytophilum***					
	1997-2003	16S rRNA	[9]	16S rRNA	[9]
	2004-2012	groEL	[10]	16S rRNA	[9]
***Borrelia burgdorferi***					
	1997-2003	p66	[14]	fla	[15]
	2004-2012	ospA	[16]	fla	[17]
***Babesia microti***					
	2002-2008	ss-rDNA	[11]	18 s rRNA	[12]
	2009-2012	ss-rDNA	[13]	18 s rRNA	[12]
***Ehrlichia muris*** **-like agent (EML)**					
	2007-2012	groEL	[10]	sequenced	

^a^Reconfirmation of samples positive in the initial screen.

### *Anaplasma phagocytophilum/Ehrlichia* spp. PCR

From 1997 through 2003, *I. scapularis* were tested for *A. phagocytophilum* using nested primers that amplify a portion of the 16S rRNA gene [8] as previously described [2]. Positive samples were confirmed using a second PCR reaction with the same assay.

From 2004 through 2012, *I. scapularis* were tested for *A. phagocytophilum* and the EML agent in a multiplex PCR for a conserved region of the *Anaplasma/Ehrlichia* groEL gene [9]. With this assay, base pair mismatches in regions of the detection probe hybridization produce species-specific melting temperature curves for organism differentiation of four tick-borne pathogens, *Ehrlichia chaffeensis*, *E. ewingii*, *A. phagocytophilum* and the EML agent [1,9]. PuRe Taq Ready-To-Go beads (GE Healthcare Life Sciences, Piscataway, NJ) were reconstituted in a 20 μl volume containing 2 μl of tick DNA as template, 0.5 μM each primer *ESp*-F and *ESp*-R, 0.2 μM probes *Ec/e*-FL, *Aph*-FL and *ESp*-RD (LightCycler-Red 640), and an additional 2.25 mM MgCl_2_ to bring the final reaction volume of MgCl_2_ to 4.05 mM. PCR was performed in a Roche LightCycler 1.2 or 2.0. (Roche Applied Science, Indianapolis, IN) using cycling parameters as described in Bell and Patel [9]. Samples positive in this initial PCR *A. phagocytophilum* were reconfirmed using the nested 16S rRNA primers from Massung et al. [8]. Positive control was nucleic acid of *A. phagocytophilum,* kindly provided by Dr. Stephen Dumler, (at that time) Johns Hopkins School of Medicine. Samples positive for the EML agent in the initial PCR were tested again in the groEL multiplex, and those positive in that test were sent to Mayo Clinic, Rochester, MN, for sequence confirmation.

The groEL multiplex that detects the EML agent was used by the HTTKP from 2004 to 2012. However, this pathogen was not described until 2009 [1], and at that time the HTTKP was able to back-check melting curve records only until 2007; therefore, only EML agent PCR results from 2007 to 2012 are reported.

### *Babesia microti* PCR

From 2002 through 2008, *I. scapularis* were tested for *Ba. microti* in a conventional PCR using universal primers targeting the ss-rDNA gene [10]. PCR was performed in 25 μL reaction volumes prepared with Ready-To-Go PCR Beads and 1 μL of tick DNA as template using cycling parameters as described. Samples positive in the initial PCR were then tested in a conventional PCR with primers targeting the 18S rRNA gene of *Ba. odocoilei* and *Ba. microti* [11]*.* PCR was performed in 25 μL reaction volumes prepared with Ready-To-Go PCR Beads and 1 μL of tick DNA using cycling parameters as described. Positive control was nucleic acid of *Ba. microti,* kindly provided by Dr. Peter Krause, (at that time) University of Connecticut School of Medicine.

From 2009 through 2012, initial screening of samples was performed in a real-time PCR using primers targeting ss rDNA of *Ba. microti* [12] prepared with Ready-To-Go beads in a 20 μl volume containing 2 μl of tick DNA and using cycling parameters as described. Samples positive in the initial PCR were then tested with the primers targeting the 18S rRNA gene of *Ba. odocoilei* and *Ba. microti* [11]. The positive control consisted of *Ba. microti* nucleic acid kindly provided by Dr. Barbara Herwaldt, Centers for Disease Control and Prevention, (CDC) Atlanta, GA.

### *Borrelia burgdorferi* PCR

From 1997 through 2003, *I. scapularis* were tested for *B. burgdorferi* in a nested PCR using primers that amplify a portion of the p66 gene [13]. Positive samples were tested with a second conventional PCR using primers targeting the fla gene of *B. burgdorferi* [14]. Use of both methods by the HTTKP has been previously described [2].

From 2004 through 2012, *I. scapularis* were tested for *B. burgdorferi* in a real-time PCR with primers and a probe for the ospA gene of *B. burgdorferi* [15] prepared with Ready-To-Go beads in a 20 μl volume containing 2 μl of tick DNA and using cycling parameters as described. Positive samples were tested by a second real-time PCR targeting the inner part of the fla gene [16] using Ready-To-Go beads in a 20 μl volume containing 2 μl of tick DNA as template and using cycling parameters as described. The positive control for both reactions was *B. burgdorferi* strain B31 (gift of Dr. Robert Wirtz, CDC Atlanta).

*B. burgdorferi* PCR of *I. scapularis* received in 2012 from Ft. McCoy and Camp Ripley was reconfirmed at Texas A&M using nested PCR for the 16S-23S rRNA intergenic spacer region [17].

### Sequencing

Sequence determination of a subset of samples from Camp Ripley found to be co-infected with one or more pathogens was performed at University of North Texas Health Science Center. Residual amplification primers were removed from PCR products using ExoSAP-IT® (USB Corporation, Cleveland Ohio) before sequence determination. DNA sequence was determined for both strands of all amplicons using a BigDye® Terminator v.3.1 Cycle Sequencing kit (Applied Biosystems, Foster City, CA). Unincorporated dye terminators were removed prior to electrophoresis using Performa® DTR Gel Filtration Cartridges (Edge BioSystems, Gaithersburg, MD). Capillary electrophoresis was performed using an ABI PRISM® 310 Genetic Analyzer (Applied Biosystems, Foster City, CA). Final sequence analysis and editing was performed using Sequencher 4.1.4 (Gene Codes Corporation, Ann Arbor MI). Pathogen identification was determined by comparison of edited DNA sequence using the BLASTN version 2.2.10 (National Center for Biotechnology Information) with sequence data in GenBank.

Sequencing was also performed at Mayo Clinic, Rochester, MN, for confirmation of a subset of EML PCR positive samples. The amplification product was purified as described above. DNA sequences for both strands of all amplicons were determined using a BigDye® Terminator v.1.1 Cycle Sequencing kit (Applied Biosystems, Foster City, CA). Unincorporated dye terminators were removed prior to electrophoresis using Performa® Ultra 96-well Plate (Edge BioSystems, Gaithersburg, MD). Capillary electrophoresis was performed using an ABI PRISM® 3730 xl DNA Analyzer (Applied Biosystems, Foster City, CA). Final sequence analysis and editing was performed using Sequencher 5.0 (Gene Codes Corporation, Ann Arbor MI). Pathogen identification was also determined as described in above paragraph.

### Pathogen prevalence and pathogen coinfection comparison

Prevalence of infection with *A. phagocytophilum*, *Ba. microti*, *B. burgdorferi* and the EML agent in *I. scapularis* adults and nymphs was compared among the three installations using pairwise Fisher’s exact tests combining across all years. Overall coinfection rates of adult and nymphal *I. scapularis* were also compared among all three sites, combining all years, using pairwise Fisher’s exact tests. To assess whether the frequency of coinfections of *A. phagocytophilum* with *B. burgdorferi*, and *Ba. microti* with *B. burgdorferi* in individual ticks differed from what would be expected due to chance under an independence assumption, an expected coinfection probability was first calculated by multiplying the individual infection probabilities. These expected probabilities were then compared to those that were observed. This was further investigated with kappa statistics to measure the degree to which the infection status agreed between the two types above and beyond chance alone.

### Investigation of the time of establishment of *I. scapularis* at Ft. Indiantown Gap

A factor which may contribute to pathogen diversity and coinfection in *I. scapularis* is the length of time that a population has been established at a given location [18]. There are few longitudinal studies of tick surveillance at Ft. Indiantown Gap, although there are numerous published studies describing ticks at Camp Ripley and Ft. McCoy. To investigate the establishment time of *I. scapularis* at Ft. Indiantown Gap over an 18-yr period, 1988-2006, HTTKP data and tick collection records from US Army entomological field studies there, and human Lyme disease case data from Lebanon Co. PA, the location of the installation, were compared.

### Phenology comparison

The relative abundance of *I. scapularis* adults vs. nymphs was compared among the three installations in the month of June, from 1997-2012, using pairwise chi-square tests. In all analyses, for each pairwise comparison between the three installations, p-values less than 0.017 were considered statistically significant using a Bonferroni adjustment. For non-pairwise comparisons, p-values of 0.05 were considered statistically significant. All analyses were performed using SAS version 9 (SAS Institute Inc., Cary, NC).

The locations of Camp Ripley, Ft. McCoy and Ft. Indiantown Gap were mapped using the calculations of the magnitude of the difference between summer and winter temperature, or the “amplitude of the annual cycle of maximum temperature” as presented in Gatewood et al. [19]. This study demonstrated that seasonal synchrony of the immature stages of *I. scapularis* is significantly related to average maximum and minimum temperatures, among other seasonal climate variables.

### Engorgement comparison

The numbers of fed (partly and fully engorged) and unengorged female *I. scapularis* were compared to the numbers of fed and unengorged nymphs collected from 1997 through 2012 using a chi-square test to investigate the presumption that tick-bite victims are more likely to detect and remove females than nymphs, due to their larger size.

This manuscript reports no research performed on human or animals. PCR testing reported throughout this article was conducted solely on ticks from the environment. The ticks had bitten humans, but they were in no way human samples. Personal Protected Information of the person bitten by the tick is not disclosed to our lab when a tick is sent for identification and testing.

## Results

### Pathogen infection

A total of 1,981 *I. scapularis* (853 adults and 1,128 nymphs) were submitted from 1997 to 2012 by Camp Ripley, Ft. McCoy and Ft. Indiantown Gap and tested for *A. phagocytophilum* and *B. burgdorferi*. Testing for *Ba. microti* was also performed on ticks received from 2002 to 2012 (632 adults and 924 nymphs) and testing for the EML agent was performed on ticks received from 2007 to 2012 (189 adult and 372 nymphs) as described above. Pathogen prevalences and ticks with evidence of polymicrobial infections are presented in Table 2. In the subset of ticks that was tested for *B. burgdorferi* at two laboratories, a 97% (69/71) concurrence was observed between the two laboratories. The Anaplasma/Ehrlichia groEL gene multiplex is designed to also detect *E. chaffeensis* and *E. ewingii;* however these agents were not detected in any *I. scapularis*.

**Table 2:** **PCR of Ixodes scapularisremoved from humans at Camp Ripley, MN, Ft. McCoy, WI and Ft. Indiantown Gap, PA for** ***Anaplasma phagocytophilum*** **,** ***Babesia microti*** **,** ***Borrelia burgdorferi*** **, and the** ***Ehrlichia muris*** **-like agent (EML)**

**Installation**	***I. scapularis***	**No. pos./no. tested (Proportion pos.)**											
		***A. phagocytophilum 1997-2012***	***Ba. microti 2002-2012***	***B. burgdorferi 1997-2012***	**EML 2007-2012**	***A.p.*** **&** ***Ba.m.***	***A.p.*** **&** ***B.b.***	***A.p.*** **& EML**	***Ba.m.*** **&** ***B.b.***	***B.b.*** **& EML**	***A.p., Ba.m.*** **&** ***B.b.***	***Ba.m., B.b.*** **& EML**	**Total co-infected**
**Camp Ripley**	Adults	49/419 (11.7%)a	6/242 (2.5%)	157/419 (37.5%)a	7/93 (7.5%)a	1/242 (0.4%)	20/419 (5%)	1/93 (1%)	1/242 (0.4%)	3/93 (3%)	1/242 (0.4%)	0	27/419 (6.4%)a
	Nymphs	26/348 (7.5%)c	7/215 (3.3%)c	90/348 (25.9%)	2/103 (1.9%)	0	12/348 (3.5%)	0	4/215 (2%)	2/103 (2%)	0	0	18/348 (5.2%)c
**Ft. McCoy**	Adults	7/201 (3.5%)b	1/175 (0.6%)	52/201 (25.9%)b	2/96 (2.1%)	0	6/201 (3%)	0	0	1/96 (1%)	0	1/96 (1%)	8/201 (4.0%)a
	Nymphs	18/480 (3.8%)	1/454 (0.2%)d	95/480 (19.8%)	0/269 (0.0%)	0	5/480 (1%)	0	0	0	0	0	5/480 (1%)d
**Ft. Indiantown Gap**	Adults	1/233 (0.4%)b	0/215 (0.0%)	82/233 (35.2%)	0/129 (0.0%)b	0	0	0	0	0	0	0	0%b
	Nymphs	5/300 (1.7%)d	0/271 (0.0%)d	59/300 (19.7%)	0/123 (0.0%)	0	0	0	0	0	0	0	0%d

Significant differences among locations are noted with superscript letters; a and b denote differences among adults, c and d denote differences among nymphs. Proportions in each column sharing different.

Superscripts were significantly different (p < 0.017). Those proportions in each column wth the same superscript were not significantly different. The absence of a superscript indicates no significant difference.

*A.p.*, *Anaplasma phagocytophilum*; *Ba.m.*, *Babesia microti*; *B.b.*, *Borrelia burgdorferi*; EML, *Ehrlichia muris*-like agent.

### Sequencing

Sequencing confirmed the presence of the suspected pathogenic agents in a subset of samples PCR-positive for polymicrobial infections and a second subset of samples positive for the EML agent. In addition, three *A. phagocytophilum* sequences, from Camp Ripley were compared with the Ap-ha 16S RNA gene sequence reported by Chen et al. [20] (GenBank accession number U02521) via BLAST sequence alignment using the optimized algorithm for highly similar sequences, Megablast. Each of these three sequences shared 100% identity with the Ap-ha sequence, the strain associated with human illness, rather than the Ap-v1 variant that is non-pathogenic to humans but common in *Odocoileus virginianus*, the white-tailed deer [21].

### Pathogen prevalence and pathogen coinfection comparison

As shown in Table 2, adult *I. scapularis* from Camp Ripley had a greater rate of *A. phagocytophilum* infection than those from Ft. McCoy (p < 0.001) and Ft. Indiantown Gap (p < 0.001) and a greater rate of *B. burgdorferi* infection than those from Ft. McCoy (p = 0.005). The EML agent was only detected from *I. scapularis* ticks submitted from Camp Ripley and Ft. McCoy and not from Ft. Indiantown Gap (p < 0.001). Nymphal *I. scapularis* from Camp Ripley had a greater rate of *A. phagocytophilum* infection than those from Ft. Indiantown Gap (p < 0.001), and a greater rate of *Ba. microti* infection than those from Ft. McCoy (p = 0.002) and Ft. Indiantown Gap (p = 0.003).

Camp Ripley and Ft. McCoy had a greater proportion of polymicrobially infected *I. scapularis* adults than Ft. Indiantown Gap (p < 0.001, p = 0.002), with Camp Ripley having a greater proportion of polymicrobially infected *I. scapularis* nymphs than Ft. McCoy (p < 0.001) and Ft. Indiantown Gap (p < 0.001) (Table 2). Slightly more coinfections of *A. phagocytophilum* and *B. burgdorferi* were observed in adults and nymphs from Ft. McCoy than would be expected under an independence assumption. There was not a statistically significant frequency of coinfection of ticks with *B. burgdorferi* and *Ba. microti* compared to what would be expected by chance.

### Time of establishment of *I. scapularis* at Ft. Indiantown Gap

Data summarized in Table 3 suggests that *I. scapularis* was not abundant at Ft. Indiantown Gap until ~ 2000. Seven studies of hunter-killed deer and small mammals and their associated ticks performed by US Army entomologists, 1988-2006, at Ft. Indiantown Gap reported an increase of *I. scapularis* and *B. burgdorferi* over these 18 years [22-25], (Melissa Miller, personal communication)*.* A corresponding increase was observed during the same time period in ticks submitted to the HTTKP, which received only *D. variabilis* from Ft. Indiantown Gap from 1993 through 1996, followed by very small numbers of *I. scapularis* in 1998 and 1999, increasing to robust populations after 2003. Human cases of confirmed and probable Lyme disease reported from Lebanon Co., PA, the location of Ft. Indiantown Gap, also show a parallel increase from 0 cases from 1980-1988, to yearly numbers of ~ 50-100 after 2003 [26].

**Table 3:** **Comparison of confirmed and probable Lyme disease cases from Lebanon Co., PA, and tick surveillance data from Ft. Indiantown Gap, Lebanon Co., PA, 1988-2006**

**Year**	**1988**	**1990**	**1991**	**1993**	**1998**	**1999**	**2000**	**2001**	**2002**	**2003**	**2004**	**2005**	**2006**
Lyme disease cases, Lebanon Co., PA	0	<5	<5	<5	5	12	13	19	36	51	80	54	46
*I. scapularis* submitted to HTTKP	0	0	0	0	1	5	7	14	18	53	13	50	46
Ticks collected from small mammals^1^	NC	NC	NC	0/156	NC	33/4	NC	NC	NC	NC	NC	NC	56/0
Ticks collected from deer^2^	0/900	3/747	0/54	NC	NC	NC	NC	NC	NC	NC	NC	84/124	NC
*B.b.* positive *I. scapularis* collected from deer	NC	2	NC	NC	NC	NC	NC	NC	NC	NC	NC	21	NC

^1^Number of *I. scapularis* collected from small mammals/number of *Dermacentor variabilis* collected from small mammals.

^2^Number of *I. scapularis* removed from hunter-killed deer/number of *Dermacentor albipictus* removed from hunter-killed deer.

NC, no collection performed.

*B.b., Borrelia burgdorferi.*

### Phenology comparison

**Figure 1:**
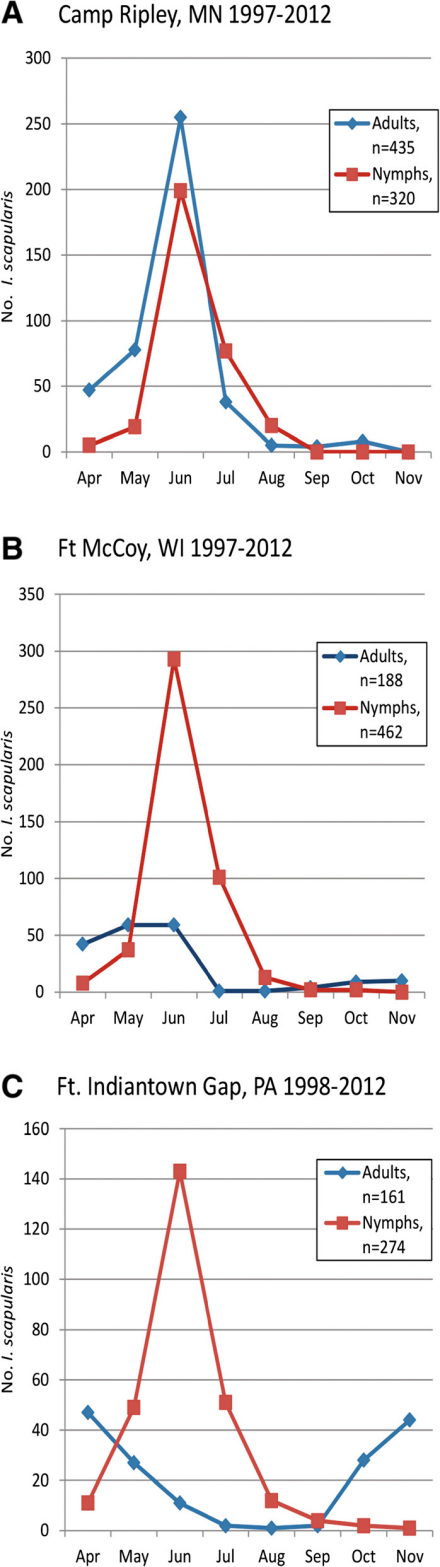
**Seasonal abundance of adult and nymphal** ***Ixodes scapularis*** **by date removed from humans. (A)** Camp Ripley, MN (adults, n=435; nymphs, n=320). **(B)** Ft. McCoy, WI (adults, n=188, nymphs, n=462). **(C)** Ft. Indiantown Gap, PA (adults, n=161; nymphs, n=274).

At all three installations, submissions of *I. scapularis* nymphs peaked distinctly in June and then declined, however, peak adult submissions varied (Figure 1). At Camp Ripley, adult and nymphal peaks overlapped in June and submissions of adults exceeded nymphs in that month. A small number of *I. scapularis* were submitted in October. At Ft. McCoy, adult tick submissions peaked in May and June, then declined until a small number of adults were submitted in October and November. At Ft. Indiantown Gap, adult submissions exhibited bimodal peaks in April and again in November.

**Figure 2:**
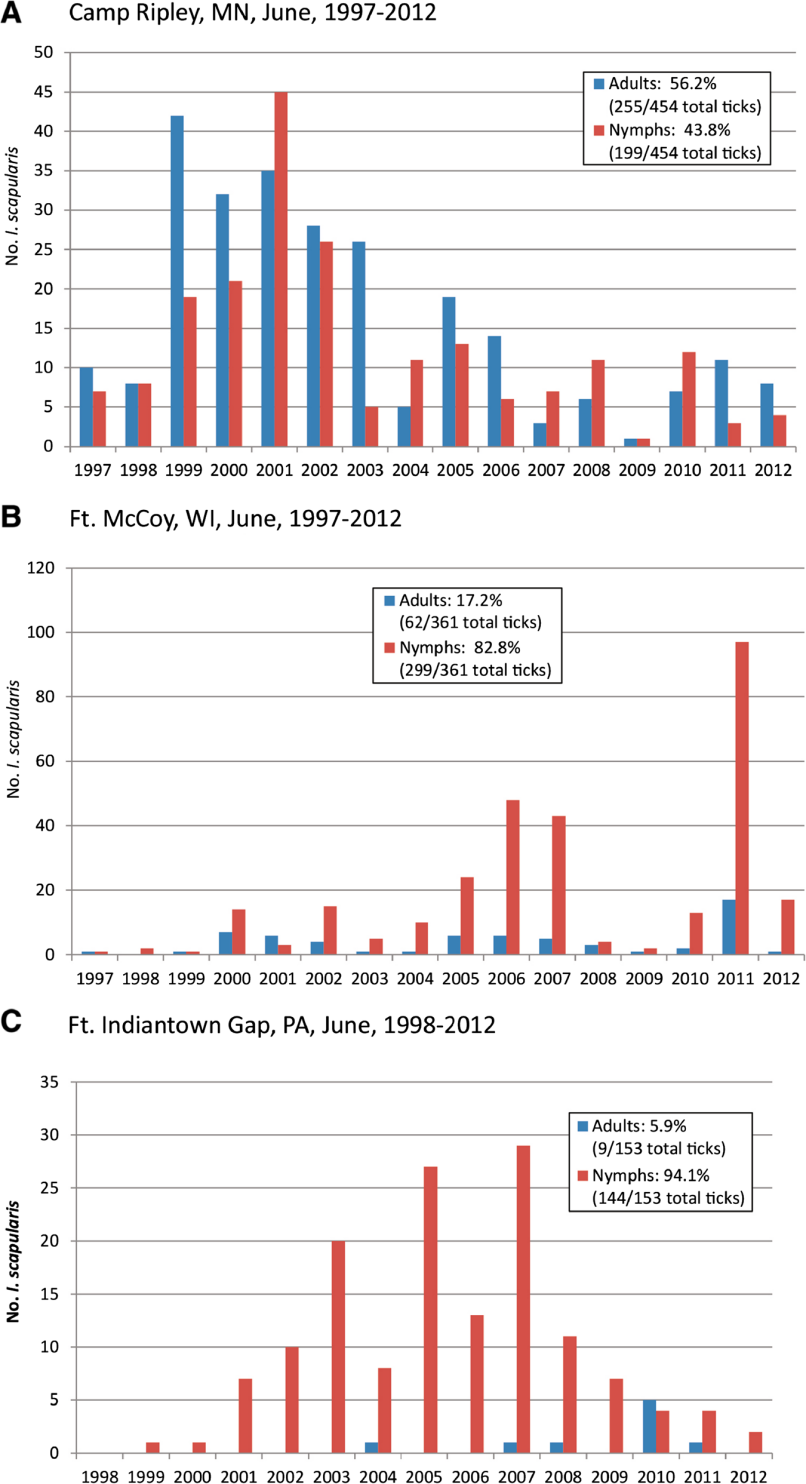
**Abundance of adult and nymphal** ***Ixodes scapularis*** **removed from humans during June. (A)** Camp Ripley, MN, adults: 56.2% (255/454 total ticks), nymphs: 43.8% (199/454 total ticks). **(B)** Ft. McCoy, WI, adults: 17.2% (62/361 total ticks), nymphs: 82.8% (299/361 total ticks). **(C)** Ft. Indiantown Gap, PA, adults: 5.9% (9/153 total ticks), nymphs, 94.1% (144/153 total ticks).

Our comparison revealed a striking difference in the relative abundance of *I. scapularis* adults vs. nymphs at the three installations in the month of June, from 1997-2012 (Figure 2). The percentage of adults was highest in June for Camp Ripley (255/454, 56.2%) as compared to Ft. McCoy (62/361, 17.2%) and Ft. Indiantown Gap (9/153, 5.9%), and these percentages were all significantly different from each other (p < 0.001 for each comparison).

**Figure 3:**
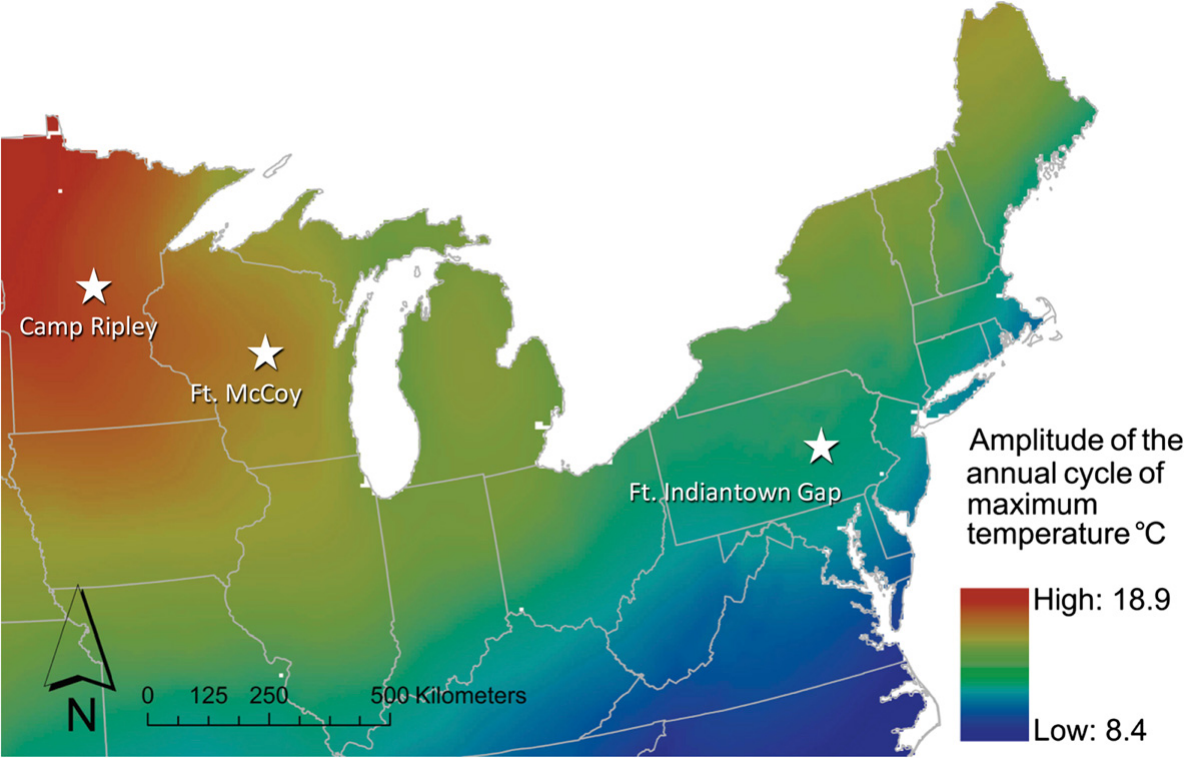
**Amplitude of the annual cycle of maximum daily temperature at Camp Ripley, MN, Ft. McCoy, WI and Ft. Indiantown Gap, PA** [28]**.** Legend: Background shading corresponds to the amplitude of the annual cycle of maximum temperature in degrees Celsius. Warm colors represent regions with extreme annual temperature cycles, cooler colors are characterized by milder seasonal climates.

Mapping the locations of Camp Ripley, Ft. McCoy and Ft. Indiantown Gap according to calculations of the magnitude of the difference between summer and winter illustrates that the amplitude of the annual cycle of maximum temperature is highest at Camp Ripley, with decreases in magnitude at Ft. McCoy, and further decreases at Ft. Indiantown Gap (Figure 3). This suggests that variation in the seasonal synchronicity of *I. scapularis* life stages – in this case, adults and nymphs - may be driven by the greater variation in seasonal climates at the different sites.

### Engorgement comparison

Overall, 91% of females and 88% of nymphal *I. scapularis* submitted to the HTTKP 1997-2012 were removed unengorged, likely reflecting daily tick checks and prompt removal of ticks by soldiers. Unattached ticks were not submitted, and <2% (127/6,678) of *I. scapularis* received by the HTTKP from 1997-2012 were males, presumably attached mating with the female ticks when removed from the human host. A small but significantly greater proportion of fed nymphs was observed compared to fed adults (142/1149 = 12.4% vs 79/871 = 9.1%, p = 0.019), supporting the presumption that tick-bite victims are more likely to detect and remove the larger female than the tiny nymph before it has time to feed and become engorged.

## Discussion

This study demonstrates clear differences in the tick phenology, pathogen prevalence and rates of co-infection among three study sites across the midwestern and northeastern US. We present evidence to support our initial observation that adult *I. scapularis* were active in the summer months in the Upper Midwest, especially at Camp Ripley, and that the EML agent was detected only in from Camp Ripley and Ft. McCoy. In this detailed comparison of our tick data from Camp Ripley, Ft. McCoy in the Upper Midwest, and Ft. Indiantown Gap in the Northeast, we also describe several other differences in prevalence of infection/coinfection of *A. phagocytophilum* and *Ba. microti*. It is likely that numerous factors and interactions are contributing to the abundance and distribution of *I. scapularis* and its associated pathogens at these sites. Several of these are discussed below with reference to several contemporary studies of environmentally-collected *I. scapularis* that investigate regional transmission cycles and examine hypotheses to explain the mechanisms driving these observed differences.

An overlap of the larval and nymphal stages of *I. scapularis* has been reported at midwestern sites, specifically Camp Ripley [5,6,19], and our data revealed an overlap of the nymphal and adult stages there, suggesting that all three life stages are active simultaneously. Gatewood et al. [19] observed that there is a greater seasonal synchrony at sites with colder temperatures, specifically, the amplitude of the annual cycle of maximum temperature. We have used a temperature gradient map from this publication to demonstrate the gradient our three study sites (Figure 3). This overlap of life stages may impact pathogen distribution. Gatewood et al. [19] hypothesized that the overlap of *I. scapularis* immature life stages in the Upper Midwest may enhance maintenance of certain short-lived strains of *B. burgdorferi*, and this phenology may also enhance maintenance of other organisms vectored by *I. scapularis*, because synchrony of life stages has been shown to favor both systemic (transmission between host and tick feeding on infectious host) and non-systemic (transmission between infected and non-infected ticks feeding in close proximity on an uninfected host) transmission [5,27]. Evidence of tick pathogen infection from our data sets indicates further differences in pathogen prevalences that may be associated with synchrony of life stages. We found greater prevalence of *A. phagocytophilum* and *Ba. microti* at *I. scapularis* from Camp Ripley and Ft. McCoy as compared to Ft. Indiantown Gap, and we detected the novel EML pathogen only at Camp Ripley and Ft. McCoy.

The EML agent was found in a small percentage of 561 *I. scapularis* adults and nymphs submitted from 2007-2012 by Camp Ripley and Ft. McCoy, but not in 252 *I. scapularis* adults and nymphs from Ft. Indiantown Gap (Table 2), nor in 1,970 *I. scapularis* adults and nymphs received by the HTTKP during the same time period and tested using the PCR methods described above from > 40 locations in Alabama, Connecticut, Delaware, Florida, Indiana, Maine, Maryland, Massachusetts, New Hampshire, New Jersey, New York, North Carolina, Pennsylvania, Rhode Island, South Carolina and Virginia (E.S., unpublished data). The presence of the EML agent in only *I. scapularis* from cold climates with seasonal synchrony of life stages suggests that these conditions may be permissive for this bacterium, and suggests that the EML agent might also be characterized by shorter periods of infectivity in vertebrate hosts.

Summer synchronous adult activity of *I. scapularis* may not play an important role in systemic pathogen maintenance in the reservoir hosts (mice, small mammals) because adult ticks are not found on these animals; adult *I. scapularis* were not collected from mice and small mammals in an extensive study of ticks at Camp Ripley [5]. However, *I. scapularis* adults also feed readily on medium-sized mammals, as do *I. scapularis* nymphs and larvae [28], so non-systemic transmission of pathogens by adults and immatures co-feeding on the larger mammal hosts may contribute to pathogen prevalence in areas where the three life stages overlap.

Recent research has revealed that length of time that a population of *I. scapularis* is present in a given location may also contribute to the pattern of pathogen diversity. Detailed studies dating from 2004 of the invasion and establishment of *I. scapularis* in lower Michigan suggested that newly-invading populations of *I. scapularis* had less pathogen diversity (specifically *A. phagocytophilum* and *Ba. microti*) but equivalent *B. burgdorferi* prevalence when compared with long-established *I. scapularis* populations in northern Michigan, Wisconsin and Minnesota [18]. Greater pathogen diversity presages coinfections in *I. scapularis*, and the pattern of pathogen infection/coinfection described from the newly-invaded as compared to the long-established Michigan sites resembles the differences in pathogen/coinfection prevalences that we observed at Ft. Indiantown Gap as compared to Camp Ripley and Ft. McCoy.

*I. scapularis* populations have been documented from Minnesota and Wisconsin for 40 years [18], and the increasing abundance and prevalence of infection of *I. scapularis* at Camp Ripley and Ft. McCoy have been the subject of surveillance since Lyme disease emerged in the late 1970s [5,29-33]. Reports of tick surveillance at Ft. Indiantown Gap are fewer but suggest that *I. scapularis* was not collected there until much more recently. Several parallel lines of inquiry suggest that *I. scapularis* established at Ft. Indiantown Gap in the late 1990s (Table 3). From these data sets, it appears that *I. scapularis* was absent or rare in the area of Ft. Indiantown Gap in the early 1990s, with numbers of ticks and cases of Lyme disease increasing in the late 1990s, then increasing sharply until reaching a plateau about 2003. Infection rates of *B. burgdorferi* were robust (3/11 = 24% in adults, 4/15 = 27% in nymphs) in ticks submitted from Ft. Indiantown Gap to the HTTKP as soon as they were first tested in 2000, but infection with *A. phagocytophilum* was rarely found in these ticks, and *Ba. microti* has not been detected (Table 2), thus supporting the hypothesis that newly-invasive populations have less pathogen diversity [18]. However, the prevalence of infection of *B. burgdorferi* was robust at all three locations, with a statistical difference in *B. burgdorferi* prevalence found only in adults from Ft. McCoy (Table 2), and other studies have reported that newly-invaded populations had nearly equivalent *B. burgdorferi* prevalences with those of long-established populations [18]. Furthermore, the duration of establishment of *I. scapularis* populations may not influence the establishment of the EML agent in these ticks, given that it has not been detected in 356 *I. scapularis* adults and nymphs submitted to the HTTKP from northeastern sites of long-established populations, Groton, CT, and Newport, RI (E.S., unpublished data).

As all ticks submitted to the HTTKP are removed from humans, this research reveals that adult *I. scapularis* at Camp Ripley are biting humans in summer alongside nymphs, and may also be important in the spread of human disease in Minnesota and other areas of synchronous tick activity. The relative abundance of adults to nymphs found biting humans in June at Camp Ripley might be explained by the size difference of adult and nymph, i.e., the larger adult is more likely to be detected; however, comparison with the June data from Ft. McCoy and Ft. Indiantown Gap, where more nymphs than adults were removed from soldiers in June, suggests that the actual distribution of adults and nymphs at that time period is accurately represented. Adult tick activity in June may have human health implications, because of the high pathogen prevalence in these ticks. Adult *I. scapularis* have higher pathogen prevalence, and a greater chance of coinfection, since the tick has completed two meals (the larval and the nymphal meal) on potentially infected hosts or alongside infected conspecifics. However, as reported above, most (91%) adult female *I. scapularis* submitted to the HTTKP were unengorged, so hopefully *B. burgdorferi* infection was forestalled in many cases by prompt tick removal, because the time of attachment of *I. scapularis* nymphs until *B. burgdorferi* transmission is thought to be >36 hours, perhaps as long as 72 hrs. [4], at which time both nymphal and adult ticks show obvious signs of engorgement [3]. The time of attachment of *I. scapularis* nymphs until transmission of *A. phagocytophilum* and *Ba. microti* in animal systems appears to be shorter, within the first 24 h of tick, when the tick does not yet appear partly engorged [3,34,35]. Transmission of Powassen virus, a life-threatening human pathogen vectored by *I. scapularis* but not investigated in the study, is thought to occur far more rapidly, within 15 min of attachment of *I. scapularis* nymphs [36]. Duration of attachment studies are lacking for novel pathogens EML and *Borrelia miyamotoi*, both associated with the bite of *I. scapularis*. Studies of the transmission of human pathogens by the adult stage of *I. scapularis* are also lacking and there is a need for better understanding of the timing from attachment until transmission occurs for the numerous pathogens vectored by this tick.

There are several potential limitations of the data collected in this study. One limitation is that the data are passively collected through detection of ticks on military personnel. The tick’s habit of attachment to the host makes it an ideal subject for passive surveillance, and the investigation of ticks removed from human hosts provides an estimate of actual human encounters with infected ticks. This information is especially important in surveillance of *I. scapularis* ticks, because in some areas of the US, *I. scapularis* is present but does not typically attack humans. However, capturing accurate temporal and spatial data of ticks removed from humans can be challenging. Ticks usually attach undetected by the host, and people often cannot accurately describe where or when a tick was acquired. Tick-bite victims are more able to recall when a tick was found and removed; therefore, we have used the date of tick removal in our phenology comparisons. Likewise, tick-bite victims may not know where they encountered their tick. Our study overcomes this limitation, in part, through our selection of three installations in which the soldiers are present only for temporary training during which they rarely leave the installations.

Another limitation is that our data were dependent on the sampling effort of the military personnel. The number of soldiers training at these installations far exceeds the numbers that have submitted ticks to the HTTKP. At Camp Ripley, for example, during April – September 2007-2011, the number of soldiers training per month was typically >10,000 (Jay Brezinka, personal communication). It is not known how many ticks fed on soldiers undetected or how many ticks were removed by soldiers and not submitted to the HTTKP. All soldiers are trained to use personal protective measures against tick-bite [37], to perform tick checks, and to submit all attached ticks to their military medical treatment facilities for identification and testing by the HTTKP, however, compliance with these instructions is thought to be inadequate [38].

## Conclusions

In summary, we demonstrate that tick phenology and pathogen prevalence exhibit statistically significant differences among three Army installations participating in the HTTKP. These observed differences are congruent with other recent studies comparing maximum seasonal temperature differences and the length of tick population establishment at these three sites. Adult *I. scapularis* ticks are biting humans in summer alongside nymphs, and may also be important in the spread of human disease in Minnesota and other areas of synchronous tick activity. Our data also support the preliminary report of Pritt et al. [1] that the EML agent appears to be limited to Minnesota and Wisconsin. Longitudinal data sets such as presented here will be useful in future studies investigating regional trends in tick species distribution and pathogen presence.
